# A Risk Prediction Model Based on Machine Learning for Cognitive Impairment Among Chinese Community-Dwelling Elderly People With Normal Cognition: Development and Validation Study

**DOI:** 10.2196/20298

**Published:** 2021-02-24

**Authors:** Mingyue Hu, Xinhui Shu, Gang Yu, Xinyin Wu, Maritta Välimäki, Hui Feng

**Affiliations:** 1 Xiangya Nursing School Central South University Changsha China; 2 Henan Cancer Hospital Province Zhengzhou University Zhengzhou China; 3 Department of Biomedical Engineering School of Basic Medical Science Central South University Changsha China; 4 Xiangya School of Public Health Central South University Changsha China; 5 Department of Nursing Science University of Turku Helsinki Finland; 6 Oceanwide Health Management Institute Central South University Changsha China; 7 National Clinical Research Center for Geriatric Disorders Xiangya Hospital Central South University Changsha China

**Keywords:** prediction model, cognitive impairment, machine learning, nomogram

## Abstract

**Background:**

Identifying cognitive impairment early enough could support timely intervention that may hinder or delay the trajectory of cognitive impairment, thus increasing the chances for successful cognitive aging.

**Objective:**

We aimed to build a prediction model based on machine learning for cognitive impairment among Chinese community-dwelling elderly people with normal cognition.

**Methods:**

A prospective cohort of 6718 older people from the Chinese Longitudinal Healthy Longevity Survey (CLHLS) register, followed between 2008 and 2011, was used to develop and validate the prediction model. Participants were included if they were aged 60 years or above, were community-dwelling elderly people, and had a cognitive Mini-Mental State Examination (MMSE) score ≥18. They were excluded if they were diagnosed with a severe disease (eg, cancer and dementia) or were living in institutions. Cognitive impairment was identified using the Chinese version of the MMSE. Several machine learning algorithms (random forest, XGBoost, naïve Bayes, and logistic regression) were used to assess the 3-year risk of developing cognitive impairment. Optimal cutoffs and adjusted parameters were explored in validation data, and the model was further evaluated in test data. A nomogram was established to vividly present the prediction model.

**Results:**

The mean age of the participants was 80.4 years (SD 10.3 years), and 50.85% (3416/6718) were female. During a 3-year follow-up, 991 (14.8%) participants were identified with cognitive impairment. Among 45 features, the following four features were finally selected to develop the model: age, instrumental activities of daily living, marital status, and baseline cognitive function. The concordance index of the model constructed by logistic regression was 0.814 (95% CI 0.781-0.846). Older people with normal cognitive functioning having a nomogram score of less than 170 were considered to have a low 3-year risk of cognitive impairment, and those with a score of 170 or greater were considered to have a high 3-year risk of cognitive impairment.

**Conclusions:**

This simple and feasible cognitive impairment prediction model could identify community-dwelling elderly people at the greatest 3-year risk for cognitive impairment, which could help community nurses in the early identification of dementia.

## Introduction

Dementia constitutes a major health care burden nationally and worldwide [1]. Approximately every 3 seconds, a person somewhere in the world is diagnosed with dementia, and the current annual cost of dementia is estimated to be US $1 trillion, which is set to double by 2030 [2]. China has the largest population of patients with dementia in the world (9.5 million) followed by the United States (4.2 million) [1]. Evidence suggests that delaying the onset of dementia by 1 year is likely to reduce its prevalence by 11% by 2050, while delaying it by 5 years could halve the number of people living with dementia by 2050 [3]. Given that dementia is incurable, it is of high importance to detect cognitive impairment in its early stages [4].

Good evidence already exists that specific risk factors can contribute to increased dementia risk at different life stages. The risk factors are education in early life, hypertension and obesity in midlife, and smoking and depression in later life [5]. Prediction models concerning risk factors for cognitive impairment have already been published. However, the variables included in the models vary, and they mostly focus on laboratory markers only [6-8]. A systematic review by Hou et al included 61 studies of the prediction models of dementia. They found that age, sex, education, cognition assessment scales, BMI, alcohol intake, and genetic variables were the most common predictors included in the models [8]. Questionnaire-based data have also been used to explore the clinical variables with promising predictive values in the transition to cognitive impairment (demographic characteristics and neuropsychiatric symptoms). Other studies have used data based on medical imaging (brain atrophy), genes (apolipoprotein Eε4), or biomarkers (amyloid-β, tau, etc) [5,9,10]. One study used the C-Pittsburgh compound B (C-PiB) medial temporal standard uptake value ratio with the Mini-Mental State Examination (MMSE) for the prediction of a person going from mild cognitive impairment to dementia, and the area under the curve was 0.92 [6]. Kivipelto et al used big data to develop a prediction model of the risk of late-life dementia in middle-aged people, and the model included age, education, hypertension, hypercholesterolemia, and obesity as variables, with an average area under the curve of 0.77 [7]. However, these prediction models are complex, less accurate, and difficult to implement in practice for nursing staff who are working with elderly patients. Therefore, especially for use in community environments, simpler, more accurate, and feasible models are needed [8].

Machine learning has recently been used to produce a prediction model for practice. Machine learning can help in modeling information based on causal and/or statistical data, potentially revealing hidden dependencies between factors and diseases in a big data environment [11]. Published studies show how machine learning algorithms, such as naïve Bayes (NB), AdaBoost, and random forest (RF), have been used to predict or detect cognitive impairment [12-15].

We systematically searched PubMed ([“cognitive impairment” OR “cognitive decline” OR “dementia” OR “alzheimer*”] AND [“machine learning” OR “data mining” OR “big data”] AND “prediction”) and found four studies in which machine learning was used to identify risk factors for dementia among people with normal cognition at baseline. One study [16] used unsupervised machine learning to develop a dementia prediction model that could identify people at a high risk of developing dementia. Another study [13] used the medical records of 93,120 patients to develop a model for exploring undetected dementia using a machine learning approach (with an area under the curve of 0.74). One study [17] developed a model for predicting the risk of developing dementia within the next 2 years among older people (aged 85 years or above) without dementia (with an area under the curve of 0.73). The study showed that the predictors differed between the youngest and oldest individuals in the population. Further, another study used supervised machine learning to develop a dementia prediction model (area under the curve values of 0.75 and 0.79) and found that the Disease State Index is useful for identifying individuals who are most at risk [18].

However, a variety of difficulties have been identified in implementing the results of machine learning in clinical practice, as the data have been collected at one time point only, meaning that the causality of the data can be questioned [13]. Some prediction models have been too complicated, and there have been problems with accuracy in the prediction [16-18]. In addition, although the results seem to be acceptable from a statistical point of view, understanding the interpretation of the unsupervised machine learning result and its implementation into practice is demanding [16]. There is still room for the improvement of prediction models for forecasting risks for dementia. In addition, more studies are needed to develop and translate the results into clinical practice, especially for community environments [19]. We therefore aimed to develop an algorithm to be used in a prediction model to identify risk factors for cognitive impairment among Chinese community-dwelling elderly people with normal cognition. The study results are important, as an approach to stratify the individual risks for cognitive impairment is needed in community settings for both national and international purposes [20].

## Methods

### Design and Participants

This study strictly followed the Transparent Reporting of a Multivariable Prediction Model for Individual Prognosis or Diagnosis (TRIPOD): The TRIPOD Statement [21]. In this machine learning approach, the national prospective longitudinal results of the Chinese Longitudinal Healthy Longevity Survey (CLHLS) were used [22]. The CLHLS is one of the largest national longitudinal studies for investigating the health of older Chinese adults. Launched in 1998, the CLHLS implemented follow-up surveys in 2000, 2002, 2005, 2008-09, 2011-12, and 2014. A total of 22 Chinese provinces were randomly recruited, and the sampling frame covered about 85% of the total population of China. The survey results in the national database are freely accessible and available online [23]. The 2008-09 survey included a total of 16,954 participants.

We included 11,788 participants from the 2008-09 wave, and 6718 participants were eligible for model development and internal validation. Participants were included if they were (1) aged 60 years or above; (2) community-dwelling elderly people; and (3) normally cognitive (MMSE score ≥18). They were excluded if they (1) were diagnosed with a severe disease (eg, cancer and dementia) or (2) lived in an institution. A detailed flow chart of participant selection is shown in Multimedia Appendix 1. Among the remaining participants, in the 2011-12 wave, a total of 1913 participants were lost in the follow-up and 2879 died. Those who were excluded from analyses owing to nonparticipation or death were on average older (*P*<.001) and had lower physical function scores (*P*<.001) and lower baseline cognition scores (*P*<.001). The two groups were not significantly different in terms of sex (*P*=.45).

### Outcome Variables and Predictors

Cognitive impairment was defined by the Chinese version of the Mini-Mental State Examination (CMMSE) [24], which was culturally translated from the international standard of the MMSE questionnaire. The CMMSE contains 24 items within six dimensions (five items for orientation, three for registration, one for naming, five for attention and calculation, three for recall, and seven for language). The score of the Chinese MMSE ranges from 0 to 30 points, with higher scores indicating better cognition. The CMMSE has been validated among the Chinese elderly population, and a score below 18 points has been defined as cognitive impairment [24].

Predictors related to cognitive impairment were assessed a priori based on clinical importance, scientific knowledge, and predictors identified in previously published studies [25]. We therefore selected 45 factors related to demographic characteristics, which included lifestyle, mental health, leisure activities, sleep, chronic diseases, physical function, anthropometric index, and baseline cognitive function (Multimedia Appendix 2).

### Statistical Analysis

Categorical variables have been reported as numbers and proportions, and compared using a chi-square test or Fisher exact test. Continuous variables have been expressed as medians with IQRs and compared using the Wilcoxon test when data were not normally distributed. Detailed information is presented in Multimedia Appendix 2. Some covariates contained missing values. The proportion of missing values was less than 5% for all variables. Thus, we performed imputations, using multivariable regression methods via the R package mice. Feature selection was performed using recursive feature elimination (RFE) combined with RF. During the process of elimination, a 10-fold cross-validation was implemented to optimize the variable selection. In addition, the RFE method with the NB method was used to extract variables, and the result was compared with RFE combined with RF. According to the results of RF and NB, the final feature selection was based on the number of features included and accuracy.

We divided the original data into a 2/3 training set, 1/6 validation set, and 1/6 test set [26]. The training set was used for model development. The validation set was used to adjust parameters of the model and explore optimal cutoffs after training was finished. The test set was used to estimate the generalization of the model. Regarding the algorithm used in the development of prediction models, we chose four machine learning algorithms, including RF, XGBoost, NB, and logistic regression, to construct models based on the results of the feature selection. We chose these four learning algorithms because they are recommended by “Guidelines for Developing and Reporting Machine Learning Predictive Models in Biomedical Research: A Multidisciplinary View” [27]. The performances of the four prediction models were compared with each other using areas under the curve, specificity, sensitivity, accuracy, and specificity/sensitivity.

In addition, if logistic regression performs well compared with the other three methods, we will formulate a nomogram based on the result of logistic regression for practical use. The nomogram works by proportionally converting each regression coefficient into a 0 to 100-point scale, with 100 points being the highest β coefficient. The points across each independent variable are added to derive total points, which are translated to predicted probabilities [28]. All analyses were conducted using R, version 3.6.0 [29]. A *P* value <0.05 was considered to indicate statistical significance.

## Results

### Population Demographics

A total of 6718 participants were involved. Forty-five explanatory variables were selected, and these variables included nine items of demographic characteristics, five items of lifestyle, 10 items of mental health, five items of leisure activities, two items of sleep, seven items of chronic diseases, two items of physical function, four items of the anthropometric index, and one item of baseline cognitive function. Six variables of demographic characteristics (age, sex, ethnicity, years of education, occupation, and marital status), four variables of lifestyle (fruit, smoking, drinking, and exercise), eight variables of mental health (self-reported quality of life, being positive, hygiene, anxiety, loneliness, decision making, feeling useless, and feeling happy), five variables of leisure activity (garden work, reading, raising pets, playing cards or mah-jongg, and social activities), two variables of sleep (sleep quality and sleep duration), five variables of chronic diseases (hypertension, diabetes, heart disease, cataract, and arthritis), two variables of physical function (activities of daily living and instrumental activities of daily living), one variable of anthropometric measurement (BMI), and baseline MMSE were significantly associated with a 3-year risk of cognitive impairment (*P*<.001). Detailed information is presented in Multimedia Appendix 2.

### Feature Selection

NB combined with RFE showed that accuracy (0.8342) was the highest with four features included in the model (age, instrumental activities of daily living, baseline MMSE, and marital status). RF combined with RFE showed that the model involving 45 variables had the highest accuracy (0.8502), while the model including four variables had an accuracy of 0.8304 (Table 1). Considering the simplicity and accuracy of the prediction model, we finally chose the following four features to develop the model: age, instrumental activities of daily living, baseline MMSE, and marital status.

**Table 1:** Feature selection using naïve Bayes combined with recursive feature elimination and random forest combined with recursive feature elimination.

Method	Number of features	Accuracy	Kappa	Accuracy SD	Kappa SD
NB^a^ combined with RFE^b^	4	0.8342	0.3258	0.007801	0.02452
NB combined with RFE	8	0.8229	0.3543	0.007340	0.02408
NB combined with RFE	16	0.8136	0.3421	0.012724	0.02540
NB combined with RFE	45	0.8315	0.3220	0.007567	0.02639
RF^c^ combined with RFE	4	0.8304	0.1545	0.008356	0.05486
RF combined with RFE	8	0.8475	0.1594	0.008545	0.05569
RF combined with RFE	16	0.8471	0.1789	0.007815	0.03612
RF combined with RFE	45	0.8502	0.1214	0.005572	0.04800

^a^NB: naïve Bayes.

^b^RFE: recursive feature elimination.

^c^RF: random forest.

### Model Evaluation and Comparison

The training, validation, and test sets involved 4514, 1100, and 1104 points of data, respectively. We tried to use several widely applied machine learning algorithms (RF, NB, XGBoost, and logistic regression) for the construction of the prediction models in the training set.

**Figure 1:**
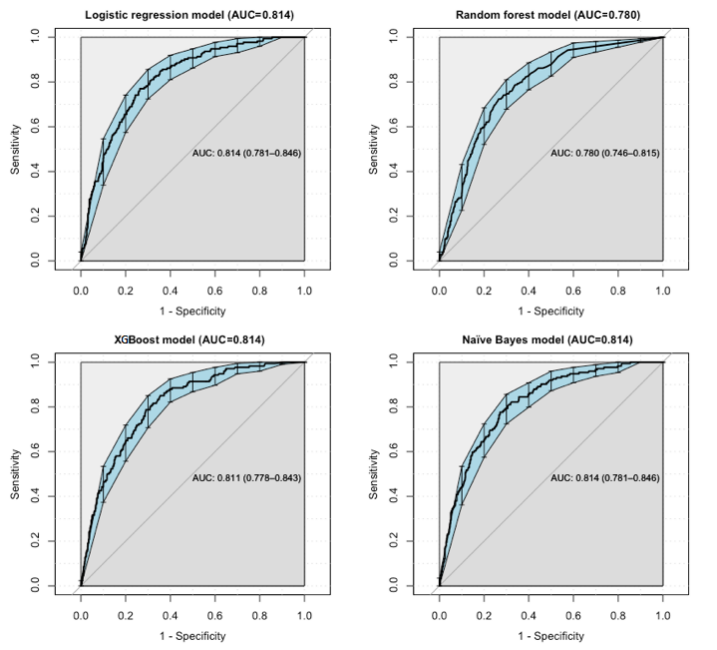
Receiver operating characteristic curve performance of four models on the test set. AUC: area under the curve.

We used a receiver operating characteristic (ROC) curve, specificity, sensitivity, accuracy, and specificity/sensitivity to evaluate the prediction model in both validation and test data. Before the evaluation, optimal cutoffs were determined by maximizing the Youden index (ie, sensitivity + specificity − 1) by the ROC curve in the validation set. In the test set, ROC curves revealed that logistic regression and NB had better predictive performances, with an area under the curve of 0.814. The area under the curve of XGBoost (0.811) was less than that of logistic regression and NB. RF underperformed, with an area under the curve of 0.780 (Figure 1).

The model of NB performed well in terms of specificity, with a value of 0.776. The specificities of the models of logistic regression (0.770), RF (0.645), and XGBoost (0.738) were lower than that of NB. The model of RF performed well in terms of sensitivity, with a value of 0.793. The sensitivities of the models of logistic regression (0.701), NB (0.672), and XGBoost (0.724) were lower than that of RF. The accuracy of the NB model (0.760) was higher than the accuracies of the other three models. All details about the parameters of the models developed with different algorithms are shown in Table 2.

**Table 2:** Evaluation of the performance of the four algorithms.

Algorithm	Data set	Area under the curve	Optimal cutoff	Specificity	Sensitivity	Accuracy	Specificity/sensitivity
Logistic regression	Validation	0.812	0.116	0.785	0.682	0.768	1.151
Logistic regression	Test	0.814	0.116	0.770	0.701	0.759	1.098
Random forest	Validation	0.773	0.040	0.654	0.784	0.675	0.834
Random forest	Test	0.780	0.040	0.645	0.793	0.669	0.813
Naïve Bayes	Validation	0.804	0.214	0.796	0.688	0.778	1.157
Naïve Bayes	Test	0.814	0.214	0.776	0.672	0.760	1.155
XGBoost	Validation	0.815	0.302	0.753	0.744	0.752	1.012
XGBoost	Test	0.814	0.302	0.738	0.724	0.736	1.019

### Development of the Nomogram

**Figure 2:**
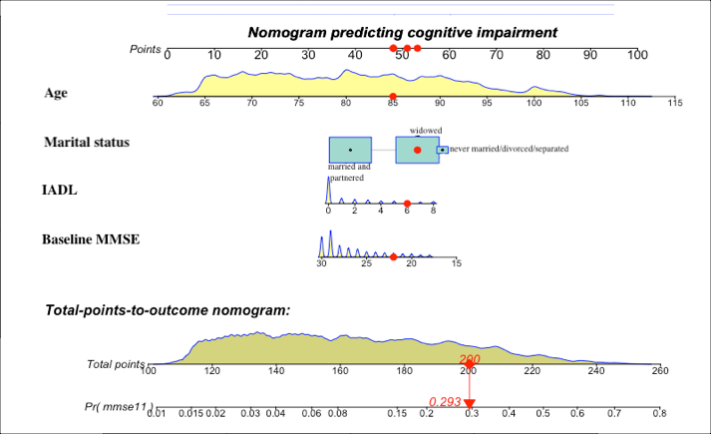
Developed nomogram with age, marital status, instrumental activities of daily living (IADL), and baseline Mini-Mental State Examination (MMSE) incorporated.

As the prediction developed by logistic regression performed well, a nomogram (Figure 2) was used to present the data and predicted probabilities vividly. The optimal cutoff value of the total nomogram scores was determined to be 170 in the test set. In Figure 2, a box has been used to represent the proportion of the sample in category variables. For instance, for marital status, most of the participants were grouped into “married or partnered” and “widowed.” Density exhibited the distribution of the sample in continuous variables. For example, for baseline MMSE, most individuals scored over 28. The total points corresponded to the predicted probability. For example, an individual score of 200 points in total, with an age score of 85, a marital status of widowed, an instrumental activities of daily living score of 6, and a baseline MMSE score of 22 corresponded to a predicted probability of cognitive impairment of 29.3%.

## Discussion

### Main Findings

In this study, we developed a prediction model for forecasting the 3-year risk of cognitive impairment among 6718 community-dwelling elderly individuals aged 60 years or older with normal cognitive function via machine learning algorithms. The model performed comparably to the best available biomarkers, such as apolipoprotein genotype [13,17], but is less expensive, easier to implement, and validated internally with reasonable results.

Feature or variable selection is central in the development of a prediction model [30]. Out of the original 45 variables, the following four variables used showed the highest accuracy of the model: age, marital status, instrumental activities of daily living, and baseline MMSE. Our findings support the previous literature regarding age [5,31], marital status [32,33], activities of daily living [34,35], and cognitive status [8,36], indicating that the predictors we selected were usable and reliable.

In this study, age was one of the predictors for the risk of cognitive impairment. It has already been estimated that, in 2020, 83% of people aged 75 years or above live with dementia in the US [37]. Those aged 65 to 74 years account for 17% of cases, while those younger than 65 years could develop dementia, but it is much less common and the prevalence is uncertain [37]. It is indicated that people aged 75 years or above should be screened for the risk of cognitive impairment. However, screening all older people for dementia is not recommended, as the benefits for that are still unclear [5]. Using large existing registers and databases can offer opportunities to explore existing information to predict the health status [38].

We found that marital status was a strong predictor for the risk of cognitive impairment. A recent meta-analysis involving 812,047 participants evaluated the association between marriage and dementia. The results showed that life-long single and widowed older people were respectively 1.42 and 1.20 times more likely to be diagnosed with dementia compared with married older people [33]. Another 10-year longitudinal population-based study including 2,288,489 individuals explored the influence of marital status and concluded that the risk of dementia in nonmarried individuals was around 1.7 times greater than that in married individuals [32]. The results might be explained by the fact that married individuals are more likely to have healthy lifestyles and participate in social activities, which might be conducive to cognitive reserve and reduced dementia risk over a lifespan [39]. On the contrary, those in widowhood might be more likely to experience a higher risk of cognitive impairment than divorced people because of the detrimental effect of stress from bereavement on hippocampal neurons or cognition. Further, as marriage has been considered a social norm, people with difficulties in communication and smaller cognitive reserves across life may be less likely to marry [33]. In today’s society, staying unmarried has become more common, and this phenomenon deserves more attention. Social factors like marital status should be taken seriously as risk determinants for cognitive impairment.

An association between physical function and cognitive capacity among older people has been found in previous studies [34,40]. Our study only included instrumental activities of daily living as a predictor for constructing the model, and it did not consider activities of daily living that represent functional ability. The reason might be that there is a natural hierarchy of functional loss associated with cognitive decline among older people [34]. Older persons with progressive cognitive decline lose the ability to perform tasks, often in the order of bathing, dressing, toileting, transferring, continence, and feeding. Therefore, older people who are not able to feed themselves might not be able to perform other tasks independently [35]. Similarly, a study by Njegovan et al found that among the 14 items of activities of daily living and instrumental activities of daily living, a hierarchy of functional items existed, with instrumental activities of daily living (such as shopping, banking, etc) being lost at higher cognitive scores than basic activities of daily living (such as eating, dressing, etc), which were lost later [34]. Our results confirmed that there was a tendency for instrumental activities of daily living to be a stronger predictor compared with activities of daily living. However, since there was overlap, subdomains of these two tools might be more meaningful for developing a prediction model. For nurses and caregivers, this information can help anticipate the need for intervention in people with declining cognition showing subtle declines in instrumental activities of daily living, which could improve the quality of life of these people and their caregivers and play an important part in health care planning [34].

Baseline cognitive function affected the degree to which cognitive scores changed over time and had a profound effect on further cognitive impairment. In one study using UK biobank data to assess the effect of baseline cognitive performance on a prediction model for 3 to 8-year risk of dementia, the results showed that cognitive performance added up to 5% (from 0.78 to 0.83) to the discriminative accuracy of the ROC model developed with the variables of age, sex, education, family history, and depression [36]. The MMSE has been the most common cognitive variable for developing a dementia prediction model [8]. However, variables of specific cognitive domains, such as memory and executive function, might be more feasible and useful predictors in constructing cognitive impairment prediction models. The total MMSE score was associated less strongly with dementia and Alzheimer disease than the episodic memory subset [8]. Therefore, future studies could consider more specific cognitive domain variables.

### Limitations

Our study has limitations, which should be considered in the interpretation of the study results. First, retained cohort members were younger and had on average better cognitive and physical functioning than those who dropped out, which can lead to studies being severely underpowered and biased toward the healthier part of the aging population. As we used a nationally representative database, the ascertainment bias could, to an extent, be limited. Second, we utilized a cross-validation approach to model development and assessment. The results still need to be validated in an independent cohort. Third, we used cohort studies with insufficient details on the duration of marriage, widowhood, and divorce to allow the exploration of a dose-response effect. A future study could take the dose-response effect of marital status on cognitive function into consideration. Fourth, the baseline MMSE was used as one of the predictors. However, other specific cognitive domains, such as memory and executive function, could also be used as features, and they might perform better than whole cognitive function. Fifth, our models were based on a prospective cohort that may have some level of bias. A prospective external validation cohort is needed for further confirmation in future research. Lastly, some predictors used in our study were measured by self-reporting, resulting in information bias. Nevertheless, self-reported data are more feasibly collected in primary health care settings, and the results can be generalized to wider communities. Despite these limitations, we believe that the results are usable in terms of cognitive impairment prevention and further intervention globally.

### Implications

This is one of the first studies where a machine learning approach has been used in a nursing context. The study showed that machine learning can be used more widely in nursing science in different contexts and various functions. The prediction models exert implications in the three-grade prevention system of diseases [38]. In the primary prevention of diseases, a cognitive impairment prediction model could provide quantitative risk value (probability) of cognitive deficiency in the next 3 years, based on the current health status, offering a more intuitive and powerful scientific tool for health education and behavioral intervention. In the secondary prevention of diseases, using noninvasive, low-cost, and easy-to-acquire variables to develop a prediction model is more practicable for staff, particularly general practitioners in community health, to bring about “early detection, early diagnosis, and early treatment,” which have large influences on medical costs for dementia. In the tertiary prevention of diseases, the prediction model could be used to predict recurrence, reducing mortality and disability [38]. A simple and feasible prediction model would also help nurses to be aware of the progression of diseases over time. Therefore, nurses could be better aware of triggers that might alarm them about any hidden problems. In addition, a precise prediction model with predictors that are more available in clinical environments could help clinical nurses understand the prognostic factors of diseases. Based on this information, nurses could offer tailored preventive interventions to patients before any signs of cognition deficits occur.

This study provides guidance for future research as well. First, the use of several algorithms to construct prediction models in specific diseases offers more opportunity to find a more suitable model with a high area under the curve and accuracy. Second, selecting the most suitable predictors is important for developing a prediction model to use in clinical practice. Easy-to-acquire, noninvasive, and low-cost variables are welcome in clinical nursing, and invasive biomarkers could improve the prediction. The former is more suitable for community health care and any clinical environment because of large populations and insufficient staff and funds, while the latter is more applicable in more specific clinical environments for people with high risk of diseases. Lastly, we included Chinese elderly people aged 60 years or above and developed a cognitive impairment prediction model. Further studies could develop cognitive impairment prediction models for middle-aged people as the World Health Organization has suggested to increase the cognitive reserve in mid-life and early aging (45-70 years) [41].

In the future, the results of this study could be used in countries and areas with less human resources, such as low- and middle-income countries, to identify elderly people with a high risk of developing cognitive deficiency in the next 3 years (ie, age, marital status, physical function, and cognitive function). Simple, relevant, and easy-to-detect risk factors would save time and resources in health care and would especially help nursing staff identify those people who are at high risk of developing cognitive impairment. As family members living with elderly people do not always recognize the early signs of dementia [42], the knowledge obtained from this study could be used to educate family members as well.

## Appendix

Multimedia Appendix 1 Flow chart of participant selection.

Multimedia Appendix 2 Detailed information about 45 features among the participants (grouped by cognitive impairment).

## Abbreviations

CLHLS: Chinese Longitudinal Healthy Longevity Survey
CMMSE: Chinese version of the Mini-Mental State Examination
MMSE: Mini-Mental State Examination
NB: naïve Bayes
RF: random forest
RFE: recursive feature elimination
ROC: receiver operating characteristic
TRIPOD: Transparent Reporting of a Multivariable Prediction Model for Individual Prognosis or Diagnosis
