# Supplementary material for: A Risk Prediction Model Based on Machine Learning for Cognitive Impairment Among Chinese Community-Dwelling Elderly People With Normal Cognition: Development and Validation Study
Source: J Med Internet Res. 2021 Feb 24;23(2):e20298. doi: 10.2196/20298 (PMC7946590; doi:10.2196/20298)
Supplement: Multimedia Appendix 1 [file jmir_v23i2e20298_app1.docx]

**Multimedia Appendix 1.** Flow chart of participant selection.

16,954 participants at baseline survey (2008-09)

11,788 participants

114 younger than 60

308 institutionalized

388 dementia

71 cancer

4285 MMSE < 18

1913 lost to follow-up

2,879 died before 2012

278 MMSE missing

6,718 participants (2011-12)
