# Supplementary material for: A Risk Prediction Model Based on Machine Learning for Cognitive Impairment Among Chinese Community-Dwelling Elderly People With Normal Cognition: Development and Validation Study
Source: J Med Internet Res. 2021 Feb 24;23(2):e20298. doi: 10.2196/20298 (PMC7946590; doi:10.2196/20298)
Supplement: Multimedia Appendix 2 [file jmir_v23i2e20298_app2.docx]

**Multimedia Appendix 2.** Detailed information about 45 features among the participants (grouped by cognitive impairment).

| **Variables** | | Cognitive impairment | |  |
| --- | --- | --- | --- | --- |
|  |  | No (5727) | Yes (991) | *p* value |
| **Demographic characteristics** | |  |  |  |
| Age |  | 78 (71, 86) | 90 (84, 95) | < 0.00 |
| Sex | Male | 2994 (52.3) | 308 (31.1) | < 0.00 |
|  | Female | 2733 (47.7) | 683 (68.9) |  |
| Residence | City/town | 2205 (38.5) | 349 (35.2) | 0.05 |
|  | rural | 3522 (61.5) | 642 (64.8) |  |
| Ethnicity | Han | 5364 (93.7) | 910 (91.8) | 0.03 |
|  | Non-Han | 363 (6.3) | 81 (8.3) |  |
| Co-residence | With household members | 4782 (83.5) | 809 (81.6) | 0.15 |
|  | alone | 945 (16.5) | 182 (18.4) |  |
| Years of education | No formal education | 2747 (48.0) | 750 (75.7) | < 0.00 |
|  | 1-5 years of education | 1620 (28.3) | 161 (16.2) |  |
|  | 6-9 years of education | 985 (17.2) | 66 (6.7) |  |
|  | 10 years of education or over | 375 (6.5) | 14 (1.4) |  |
| Occupation | Agriculture/housework | 4085 (71.3) | 822 (82.9) | < 0.00 |
|  | Non-agriculture | 1642 (28.7) | 169 (17.1) |  |
| Economic status | Rich | 820 (14.3) | 127 (12.8) | 0.11 |
|  | Fair | 3993 (69.7) | 682 (68.8) |  |
|  | Poor | 914 (16.0) | 182 (18.4) |  |
| Marital status | Married and living with spouse | 2963 (51.7) | 196 (19.8) | < 0.00 |
|  | Widowed | 2570 (44.9) | 768 (77.5) |  |
|  | Never married‎/divorced‎/separated | 194 (3.4) | 27 (2.7) |  |
| **Lifestyles** | | | | |
| Fruit (How often do you eat fresh fruit?) | Almost everyday | 763 (13.3) | 115 (11.6) | 0.02 |
|  | Quite often | 1630 (28.5) | 243 (24.5) |  |
|  | Occasionally | 2128 (37.2) | 397 (40.1) |  |
|  | Rarely or never | 1206 (21.1) | 236 (23.8) |  |
| Vegetables (How often do you eat fresh vegetables?) | Almost everyday | 3856 (67.3) | 639 (64.5) | 0.19 |
|  | Quite often | 1404 (24.5) | 259 (26.1) |  |
|  | Occasionally | 391 (6.8) | 82 (8.3) |  |
|  | Rarely or never | 76 (1.3) | 11 (1.1) |  |
| Smoking (Do you smoke or not at present?) | Never | 3438 (60.0) | 744 (75.1) | < 0.00 |
|  | Former | 914 (16.0) | 126 (12.7) |  |
|  | Current | 1375 (24.0) | 121 (12.2) |  |
| Drinking (Do you drink or not at present?) | Never | 3669 (64.1) | 746 (75.3) | < 0.00 |
|  | Former | 776 (13.5) | 116 (11.7) |  |
|  | Current | 1282 (22.4) | 129 (13.0) |  |
| Exercise (Do you exercise or not at present?) | Never | 3030 (52.9) | 637 (64.3) | < 0.00 |
|  | Former | 539 (9.4) | 110 (11.1) |  |
|  | Current | 2158 (37.7) | 244 (24.6) |  |
| **Mental health** | | | | |
| Self-reported quality of life | Very good | 838 (14.6) | 134 (13.5) | 0.00 |
|  | Good | 2585 (45.1) | 497 (50.2) |  |
|  | Fair | 1957 (34.2) | 283 (28.6) |  |
|  | Bad | 311 (5.4) | 66 (6.7) |  |
|  | Very bad | 36 (0.6) | 11 (1.1) |  |
| Self-reported health | Very good | 808 (14.1) | 112 (11.3) | 0.07 |
|  | Good | 2307 (40.3) | 421 (42.5) |  |
|  | Fair | 1846 (32.2) | 317 (32.0) |  |
|  | Bad | 707 (12.3) | 125 (12.6) |  |
|  | Very bad | 59 (1.0) | 16 (1.60 |  |
| Being positive (Do you always look on the bright side of things?) | Always | 827 (14.4) | 125 (12.6) | 0.03 |
|  | Often | 3828 (66.8) | 640 (64.6) |  |
|  | Sometimes | 838 (14.6) | 177 (17.9) |  |
|  | Seldom | 208 (3.6) | 42 (4.2) |  |
|  | Never | 26 (0.5) | 7 (0.7) |  |
| Hygiene (Do you keep my belongings neat and clean?) | Always | 839 (14.6) | 123 (12.4) | 0.01 |
|  | Often | 3490 (60.9) | 586 (59.1) |  |
|  | Sometimes | 1318 (23.0) | 263 (26.5) |  |
|  | Seldom | 74 (1.3) | 15 (1.5) |  |
|  | Never | 6 (0.1) | 4 (0.4) |  |
| Anxiety (Do you feel fearful or anxious?) | Always | 92 (1.6) | 18 (1.8) | < 0.00 |
|  | Often | 226 (3.9) | 40 (4.0) |  |
|  | Sometimes | 1080 (18.9) | 248 (25.0) |  |
|  | Seldom | 1908 (33.3) | 324 (32.7) |  |
|  | Never | 2421 (42.3) | 361 (36.4) |  |
| Loneliness (Do you feel lonely and isolated?) | Always | 95 (1.7) | 22 (2.2) | < 0.00 |
|  | Often | 283 (4.9) | 63 (6.4) |  |
|  | Sometimes | 1064 (18.6) | 261 (26.3) |  |
|  | Seldom | 1731 (30.2) | 106 (30.9) |  |
|  | Never | 2554 (44.6) | 339 (34.2) |  |
| Decision-making (Do you make own decision?) | Always | 2237 (39.1) | 264 (26.6) | < 0.00 |
|  | Often | 1600 (27.9) | 216 (21.8) |  |
|  | Sometimes | 1212 (21.2) | 284 (28.7) |  |
|  | Seldom | 485 (8.5) | 166 (16.8) |  |
|  | Never | 193 (3.4) | 61 (6.3) |  |
| Feeling useless ( Do you feel useless with age?) | Always | 338 (5.9) | 88 (8.9) | < 0.00 |
|  | Often | 919 (16.0) | 178 (18.0) |  |
|  | Sometimes | 1917 (33.5) | 371 (37.4) |  |
|  | Seldom | 1270 (22.2) | 212 (21.4) |  |
|  | Never | 1283 (22.4) | 142 (14.3) |  |
| Feeling happy (Do you feel happy as younger?) | Always | 462 (8.1) | 76 (7.7) | 0.00 |
|  | Often | 1089 (19.0) | 174 (17.6) |  |
|  | Sometimes | 2006 (35.0) | 301 (30.4) |  |
|  | Seldom | 1565 (27.3) | 312 (31.5) |  |
|  | Never | 605 (10.6) | 128 (12.9) |  |
| Feeling health change (Do you feel any change of your health since last year?) | Much better | 133 (2.3) | 20 (2.0) | 0.42 |
|  | Better | 518 (9.0) | 79 (8.0) |  |
|  | No change | 3228 (56.4) | 544 (54.9) |  |
|  | A little worse | 1634 (28.5) | 309 (31.2) |  |
|  | Much worse | 214 (3.7) | 39 (3.9) |  |
| **Leisure activity** | | | | |
| Garden work (Do you do garden work?) | Almost everyday | 745 (13.0) | 53 (5.3) | < 0.00 |
|  | Once a week | 146 (2.5) | 14 (1.4) |  |
|  | Once a month | 94 (1.6) | 9 (0.0) |  |
|  | Sometimes | 154 (2.7) | 14 (1.4) |  |
|  | Never | 4588 (80.1) | 901 (90.9) |  |
| Reading (Do you read newspapers/books at present?) | Almost everyday | 788 (13.8) | 42 (4.2) | < 0.00 |
|  | Once a week | 255 (4.5) | 21 (2.1) |  |
|  | Once a month | 169 (3.0) | 13 (1.3) |  |
|  | Sometimes | 253 (4.4) | 22 (2.2) |  |
|  | Never | 4262 (74.4) | 893 (90.1) |  |
| Raising pets (Do you raise domestic animals/pets at present?) | Almost everyday | 1536 (26.8) | 166 (16.8) | < 0.00 |
|  | Once a week | 197 (3.4) | 43 (4.3) |  |
|  | Once a month | 98 (1.7) | 19 (1.9) |  |
|  | Sometimes | 172 (3.0) | 34 (3.4) |  |
|  | Never | 3724 (65.0) | 729 (73.6) |  |
| Playing cards or mah-jongg (Do you play cards/mah-jongg at present?) | Almost everyday | 505 (8.8) | 41 (4.1) | < 0.00 |
|  | Once a week | 344 (6.0) | 28 (2.8) |  |
|  | Once a month | 189 (3.3) | 16 (1.6) |  |
|  | Sometimes | 289 (5.0) | 23 (23) |  |
|  | Never | 4400 (76.8) | 883 (89.1) |  |
| Social activities (Do you take part in some social activities at present?) | Almost everyday | 227 (4.0) | 14 (1.4) | < 0.00 |
|  | Once a week | 140 (2.4) | 15 (1.5) |  |
|  | Once a month | 227 (4.0) | 25 (2.5) |  |
|  | Sometimes | 403 (7.0) | 30 (3.0) |  |
|  | Never | 4730 (82.6) | 907 (91.5) |  |
| **Sleep** |  |  |  |  |
| Sleep duration (h) (How long do you sleep normally?) |  | 8 (6, 9) | 8 (7, 10) | < 0.00 |
| Sleep quality (How about the quality of your sleep?) | Very good | 850 (14.8) | 145 (14.6) | 0.04 |
|  | Good | 2988 (52.2) | 519 (52.4) |  |
|  | Fair | 1295 (22.6) | 252 (25.4) |  |
|  | Bad | 549 (9.6) | 67 (6.8) |  |
|  | Very bad | 45 (0.8) | 8 (0.8) |  |
| **Chronic disease** | | | | |
| Hypertension (Do you suffer from hypertension?) |  | 1319 (23.0) | 195 (19.7) | 0.02 |
|  |  | 4408 (77.0) | 796 (80.3) |  |
| Diabetes (Do you suffer from diabetes?) |  | 191 (3.3) | 11 (1.1) | < 0.00 |
|  |  | 5536 (96.7) | 980 (98.9) |  |
| Heart disease (Do you suffer from heart disease?) |  | 567 (9.9) | 71 (7.2) | 0.01 |
|  |  | 5160 (90.1) | 920 (92.8) |  |
| Stroke or cardiovascular diseases (Do you suffer from stroke or cardiovascular diseases?) |  | 296 (5.2) | 55 (5.5) | 0.62 |
|  |  | 5431 (94.8) | 936 (94.5) |  |
| Lung diseases (Do you suffer from bronchitis, emphysema, pneumonia, or asthma?) |  | 568 (9.9) | 84 (8.5) | 0.16 |
|  |  | 5159 (90.1) | 907 (91.5) |  |
| Cataract (Do you suffer from cataract?) |  | 471 (8.2) | 124 (12.5) | < 0.00 |
|  |  | 5256 (91.8) | 867 (87.5) |  |
| Arthritis (Do you suffer athrisis?) |  | 1350 (23.6) | 198 (20.0) | 0.01 |
|  |  | 4377 (76.4) | 793 (80.0) |  |
| **Physical function** | | | | |
| ADL^a^ |  | 0 (0, 0) | 0 (0, 0) | < 0.00 |
| IADL^b^ |  | 0 (0, 2) | 3 (1, 6) | < 0.00 |
| **Anthropometric index** | | | | |
| BMI^c^ |  | 20.8 (18.7, 23.3) | 19.6 (17.8, 22.0) | <0.00 |
| Systolic blood pressure |  | 134.0 (123.0, 150.0) | 132.5 (122.8, 149.0) | 0.29 |
| Diastolic blood pressure |  | 79.0 (72.0, 85.5) | 80.0 (71.5, 86.0) | 0.97 |
| Heart rate |  | 73.0 (68.5, 78.5) | 73.5 (68.5, 78.5) | 0.39 |
| **Cognitive function** | | | | |
| Baseline MMSE^d^ |  | 28 (26, 29) | 26 (22, 28) | <0.00 |

Abbreviations: ^a^ ADL, activities of daily living; ^b^ IADL, instrumental activities of daily living; ^c^ BMI, body mass index; ^d^ MMSE, mini-mental state examination.
